# Revisiting revitalization: exploring how structural determinants moderate pathways between neighborhood change and health

**DOI:** 10.1186/s12939-022-01771-9

**Published:** 2022-11-18

**Authors:** Yeeli Mui, Gabby Headrick, Jessie Chien, Craig Pollack, Haneefa T. Saleem

**Affiliations:** 1 Department of International Health, Johns Hopkins Bloomberg School of Public Health, Baltimore, USA; 2 Department of Community Health Sciences, UCLA Fielding School of Public Health, Los Angeles, USA; 3 Department of Health Policy and Management, Johns Hopkins Bloomberg School of Public Health, Baltimore, USA

## Abstract

Vacant housing can produce many issues that affect residents’ quality of life, especially in historically segregated communities of color. To address these challenges, local governments invest in strategic, place-based revitalization initiatives focused on the regeneration of vacant housing. Yet, the outcomes of these efforts remain contested. To maximize health benefits of revitalization investments, a more nuanced understanding of pathways between neighborhood changes and residents’ responses, adaptations, and ability to thrive is necessary, though, remains largely absent in the literature. Using the Vacants to Value initiative in Baltimore, MD as a case study, we explore (1) how health manifests among certain groups in the context of vacant housing revitalization; (2) how vacant housing and its regeneration engender social and cultural environmental change i.e., gentrification; and (3) what structural determinants (cultural norms, policies, institutions, and practices) contribute to the distribution of material resources and benefits of revitalization. Results suggest that vacant housing revitalization requires more than just physical remedies to maximize health. Our findings demonstrate how vacant housing revitalization influences the physical environment, social environment, and structural determinants of material resources and community engagement that can ultimately impact residents’ physical, mental, and social health. This study recommends that because housing disparities are rooted in structural inequalities, how policies, practices, and processes moderate pathways for residents to adapt and benefit from neighborhood changes is consequential for health and health equity. Establishing shared governance structures is a promising approach to foster equitable decision-making and outcomes. Going forward in urban regeneration, pathways to retain and strengthen the social environment while revitalizing the physical environment may be promising to achieve healthy communities.

**Supplementary information:**

The online version contains supplementary material available at 10.1186/s12939-022-01771-9.

## Introduction

Urban inequalities in housing – including vacant housing – have important implications for health and health disparities (Cohen et al. 2003; Hernandez and Swope 2019; Krieger and Higgins 2002). Vacant housing has been shown to contribute to premature mortality from chronic illnesses, poor mental health, diminishing surrounding property values, engendering crime, and creating fire hazards (Branas et al. 2012; Garvin et al. 2013; Han 2014; Mansfield et al. 1999; Schachterle et al. 2012). Today, vacant housing in the United States is more likely to be concentrated in higher-poverty communities of color, where historical segregation and discriminatory housing policies have cemented conditions such that a greater share of Black and Latinx residents live in neighborhoods that are under-resourced and more socially vulnerable (Wang and Immergluck 2018).

Following the foreclosure crisis, vacancies in the United States reached a peak at an estimated 12 million in 2010 (Mallach 2018). Since then, cities across the country have focused on housing and community development as a key policy instrument to improve urban health by directing resources to rehabilitate, restore, or regenerate housing and neighborhoods in disrepair. However, outcomes of such “revitalization” projects remain contested. This is in part due to the variety of stakeholders who are involved and can be affected – from residents to public and private actors – and the many ways through which vacant housing revitalization may lead to a spectrum of outcomes that ultimately impact residents’ health.

Extensive literature in community development, housing, and planning has demonstrated some benefits from revitalization with increased access and availability of resources and opportunities in neighborhoods, such as better housing, walkability, urban greening, and increases in sales prices of surrounding properties (Baird et al. 2020; Schilling and Logan 2008; Wilson et al. 2008; Kondo et al. 2018; Leyden 2003; Zielenbach and Voith 2010. Still, there is often concern that revitalization will lead to gentrification. While the definition, causes, and consequences of gentrification have been widely debated, it is commonly described as a sociocultural and structural process whereby neighborhoods that have seen decades of disinvestment experience demographic change and distinct shifts “in neighborhood-level affluence, marked by higher housing costs, changes in neighborhood amenities…ultimately leading to an increased cost of living in an area” (Cole et al. 2017; Jelks et al. 2021; Kennedy and Leonard 2001; Schnake-Mahl et al. 2020). More recent studies show differential benefits of revitalization that may promote the health of more privileged residents while harming or not benefiting the health of underprivileged residents (Ehrenfeucht and Nelson 2013). Indeed, to maximize health benefits of revitalization investments and reduce health inequalities, opportunities for residents to respond, adapt, and thrive alongside neighborhood changes is necessary (Bélanger 2012; Mehdipanah et al. 2015; Cole et al. 2021). Yet, the nuances in how health manifests among certain groups in the context of vacant housing revitalization, including dynamics between “stayers” (i.e., long-term residents) and new arrivals, remains largely absent in the literature.

Building on existing theoretical frameworks described below, this study investigates the relationship between vacant properties, its revitalization process, and reports of how revitalization pathways may moderate environmental changes and residents’ responses in ways that de(stabilize) health outcomes. In our case, revitalization is taking place through the regeneration of vacant housing. An exploratory case study was conducted in Baltimore, MD, where neighborhoods across the city are experiencing revitalization under the purview of the Vacants to Value initiative. The next section presents a review of the literature on neighborhood change and health, followed by the conceptual framework guiding this study and a presentation of the case study. Section Method presents the methodological approach, including site selection, recruitment of key informant, in-depth interviews, and qualitative data analysis. Results on the structural determinants of physical, mental, and social health in the setting of vacant housing revitalization are discussed in Sect. Result. This paper concludes with a discussion in Sect. Discussion of health benefits associated with improved housing structures, perceived safety, and access to green space that were ultimately diluted by detriments to residents’ social and cultural environments, particularly experienced by long-term residents.

## Background

### Neighborhoods and the built environment as a social determinant of health

The condition of homes and their surrounding neighborhood environment is a key social determinant of health and health equity, for individuals occupying those homes as well as residents nearby (Hernandez and Swope 2019). Vacant housing, which is often clustered in urban settings, can produce many issues that affect the quality of life of residents. Vacant housing has been associated with crime, social isolation, pre-mature mortality, and a range of negative health outcomes, from cardiovascular disease and asthma to poor mental health (Accordino and Johnson 2000; Cohen et al. 2003; Garvin et al. 2013; Spelman 1993; Wang and Immergluck 2018). Additionally, vacant housing significantly impacts the fiscal conditions and resources of municipalities through the loss of tax revenues and the substantial costs incurred by local governments to manage vacant properties, including police, fire, and public health departments, code enforcement, and public works (Mallach 2018; The National Vacant Properties Campaign, 2005). Ultimately, negative externalities of vacant housing can create reinforcing feedback loops that amplify vacancy rates and related community health challenges (Immergluck 2016).

### Structural determinants of vacant housing

Population change is a strong predictor of vacant housing, and cities that have the highest vacancy rates today are those that have undergone the most drastic population declines in recent decades (Newman et al. 2019). For example, Baltimore, MD experienced the highest absolute population loss from 1990 to 2000 by approximately 85,000 individuals or -11.5% of the population (Cohen 2001) and is among the top four cities in the U.S. with the highest absolute population loss from 1950 to 2000 by almost 300,000 individuals or -31.4% of the population. In 2010, Baltimore had an estimated population of 620,000 and 31,000 vacant properties (Jacobson 2015).

The World Health Organization (WHO) defines structural determinants as the cultural norms, policies, institutions, and practices that generate or reinforce individual socioeconomic position and “configure the health opportunities of social groups based on their placement within hierarchies of power, prestige and access to resources” (Solar and Irwin 2010). Robust scholarship has developed over the last several decades on neighborhood change in U.S. depopulating, or shrinking cities, focusing in large part on key structural determinants of unequal demographic and socioeconomic shifts (Cohen 2001). These drivers comprise global economic restructuring and deindustrialization of cities as well as federal policies and spending programs that subsidized mostly white middle class out-migration from cities. Taking a closer look within cities, discriminatory lending practices that redlined certain neighborhoods isolated Black Americans from housing and economic opportunities (Ehrenfeucht and Nelson 2020). The confluence of these factors has resulted in significant population declines and subsequent increases in vacant housing, particularly in segregated pockets of cities where decades of disinvestment continue to shape concentrated social, economic, and health disadvantage today (Ehrenfeucht and Nelson 2013; Hackworth 2018).

### Mixed outcomes of policy efforts to address vacant housing

To improve quality of life and address neighborhood detriments related to vacant housing, local governments in the U.S. and abroad have invested millions of dollars in strategic, place-based revitalization initiatives, many of which center on demolishing or regenerating vacant housing to make them habitable (Herstad 2017; McGovern 2006). The effects of these efforts on the health and wellbeing of residents, however, remain contested. Unfortunately, gaining more recent attention are some of the negative consequences of revitalization initiatives, one key area of concern being gentrification. Gentrification has prompted intense academic debate over its manifestations and impact on health inequalities (Cole et al. 2021; Bhavsar et al. 2020; Gibbons et al. 2018). Much of the research points to benefits of gentrification comprising the introduction of new material resources and improved conditions, such as green space, walkability, cleaner streets, and safety. Yet, these benefits of gentrification are counterbalanced by other factors, including exacerbated social, cultural, and economic exclusion as well as food insecurity (Cole et al. 2017; Anguelovski 2015).

Whether revitalization has a net positive or negative impact on the health of communities remains inconclusive for several reasons. First, this is in large part due to variation in the literature regarding both how revitalization is defined and the wide range of contexts, scale, and tools that are utilized (Hyra 2012). Second, research examining the pathways between vacant housing, its regeneration, and health is not well understood, mainly relying on improvements in the built environment, higher property values, and increased tax revenues for municipalities (Lee 2021). The level of social inclusion in revitalization processes, for example, may have consequences related to health by affecting the allocation of resources and gains from socioeconomic developments (Tolib et al. 2022). Third, while revitalization does not always displace original residents of formerly impoverished neighborhoods, neighborhood change can impact the health of long-term residents by shifting the trajectory of the community and enabling gentrification, though how interactions between long-term residents and new arrivals influence health is also underexplored.

### Theoretical framework

**Fig. 1:**
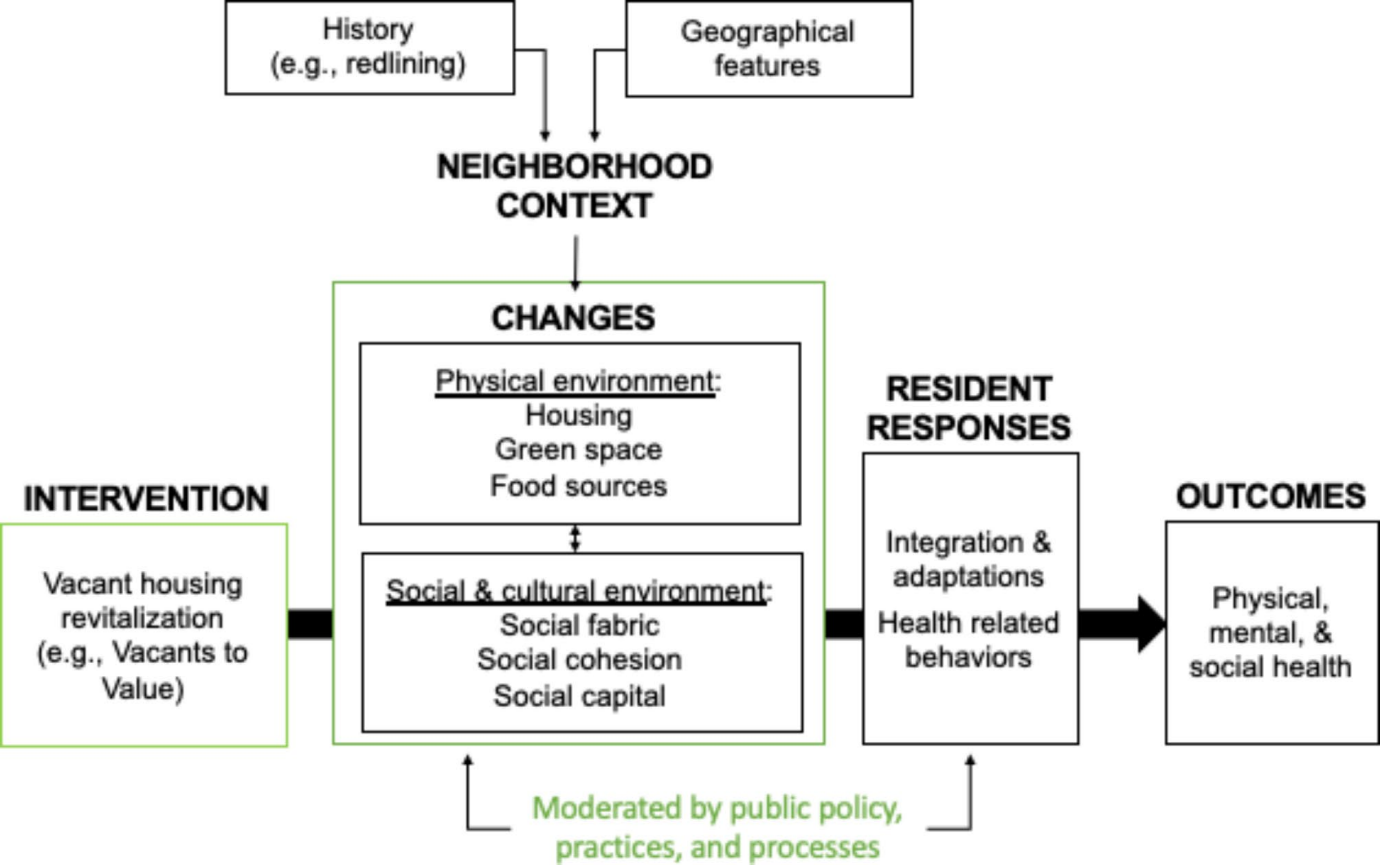
Conceptual framework of vacant housing revitalization and health moderated by structural determinants (in green) i.e., public policy, practices, and processes. (adapted from Bhavsar et al. 2020; Mehdipanah et al. 2015)

Our study draws from the Commission on Social Determinants of Health conceptual framework by the WHO and a review of current literature (Bhavsar et al. 2020; Mehdipanah et al. 2015), which describe how revitalization pathways through interventions like the regeneration of vacant housing may moderate neighborhood changes as well as residents’ responses and adaptation to neighborhood changes in ways that impact health (Fig. 1). This remains a challenge for city officials and housing practitioners. For example, while improved neighborhood safety is often attributed to neighborhood revitalization (Kreager et al. 2011), emerging research on neighborhood change and crime suggest a positive correlation between increased income inequality and crime (Metz and Burdina 2016). Further, a qualitative study from the *Turning the Corner* project, aiming to monitor neighborhood change and support displacement prevention, reported on the complexity of how residents perceive public safety beyond crime rates, including connection with neighborhoods and a sense of community (Cohen et al. 2019). The social environment has been linked to mental health disparities, and more recent studies on urban renewal and gentrification have been associated with cultural displacement, anxiety, and poor health, especially among Black individuals, low-income populations, and legacy residents (Ellen et al. 2020; Mama et al. 2016; Mehdipanah et al. 2018; Smith et al. 2020). Altogether, approaching vacant housing revitalization from a structural determinants of health perspective raises a series of salient questions:

1. How does health manifest among certain groups in the context of vacant housing revitalization?
2. How does vacant housing revitalization engender social and cultural environmental change?
3. What structural determinants (cultural norms, policies, institutions, and practices) are involved in the distribution, or maldistribution, of material resources and benefits of vacant housing revitalization?

Our study sought to investigate these questions among stakeholders, including residents, local government officials, and developers affected by the Vacants to Value initiative in Baltimore, MD.

### Case study of Baltimore: vacants to value

Baltimore, MD is one of the oldest industrial centers and port cities in the country. It serves as an ideal candidate to explore our research questions given its extended history of urban regeneration and revitalization dating back to the 1960s. Central to Baltimore’s architecture are row homes, which comprise more than half of the city’s housing stock (Hollander et al. 2019). Baltimore remains one of America’s most segregated cities (Massey 2015), with an estimated population of 620,000 and 31,000 vacant properties in 2010 (Jacobson 2015). In the same year, the city launched the Vacants to Value (V2V) initiative to revitalize vacant properties into productive use in Baltimore’s middle-market neighborhoods. Overseen by the Department of Housing and Community Development (DHCD), in partnership with the Housing Authority of Baltimore City, V2V enabled local government to work with private owners, nonprofit, and for-profit developers and to reduce the number of vacant properties by utilizing seven core strategies. These strategies included: (i) streamlining the disposition of City-owned properties; (ii) streamlining code enforcement and the use of receivership; (iii) investing in emerging markets; (iv) providing home buying incentives; (v) supporting ongoing large scale redevelopment; (vi) demolishing properties in severely distressed areas; and (vii) providing support for home improvements to owner and renter occupied homes (Jacobson 2015). Broadly, city officials describe V2V as a code enforcement strategy to lead to the elimination of blighted properties . A central element of V2V was to direct specific strategies to specific types of areas in the city based on market criteria, known as Community Development Clusters (hereafter referred to as Clusters). In Clusters characterized by high vacancy but proximity to areas with stronger and more stable market conditions, the city focused on demolition and supporting developers in large-scale redevelopment. In other Clusters characterized by a few scattered vacant homes and strong market conditions, the city focused on code enforcement to motivate homeowners to rehabilitate or sell their vacant properties. DHCD also partnered with the Office of Sustainability to facilitate greening of vacant lots created through demolition (Mallach 2017).

To date, three evaluation studies have documented the outcomes of V2V, with mixed findings. The initiative was found to be successful in eliminating blighted properties in some focus areas by renovating many previously abandoned homes and vacant lots (BNIA, 2016; Jacobson 2015; Mallach 2017). Over 1,500 properties were listed as “completed” in the first five years of the initiative, meeting the initiative’s goal for this timeframe (Jacobson 2015). However, reports of drawbacks included the total time from citing a negligent homeowner to transferring the property to a developer, which could take upwards of three years on average; poor communication of program goals between developers and communities; and limited inclusion of low-income community members in planning (BNIA 2016; Jacobson 2015; Mallach 2017). In addition, while reporting to track V2V’s participation and progress on vacant properties has faced challenges in regularity and transparency, one report found that nearly twice as many houses on the city’s list of completed homes were investor-owned (64%) as were owner occupied (34%) – a pattern consistent across the city showing a larger share of restored homes purchased by private investors relative to homeowners (Jacobson 2015). Overall, findings from these evaluation studies underscore an incomplete understanding of the structural determinants of health in the setting of vacant housing revitalization.

## Methods

### Site selection

We used a case study approach (Yin and Campbell 1984) to retrospectively explore our research questions. Given our research questions and goal of understanding revitalization processes in varied contexts, we consulted with DHCD who provided guidance on identifying Clusters in three distinct neighborhoods of northwest, east, and central Baltimore that varied in neighborhood footprint, degree of vacancy, racial diversity, and urban form (Table 1). Located furthest from city center, Cluster A was the largest in geographic size relative to Clusters B and C and had a slightly higher degree of vacancy, weaker housing market, and a lower mix of amenities at baseline (i.e., before V2V). Revitalization efforts in this cluster focused primarily on supporting large-scale redevelopment and demolition of severely distressed blocks. Cluster B comprised about nine city blocks and was situated within a smaller neighborhood that was characterized as relatively high in terms of the degree of vacancy and moderate regarding the mix of amenities and racial diversity among residents, compared to Clusters A and C. Here, V2V strategies focused on facilitating investment in an “emerging market” and targeting home-buying incentives. Cluster C exhibited the strongest current day housing market of the three clusters with a greater mix of amenities, including close proximity to transportation options and a growing arts scene; this Cluster was also beginning to experience mounting pressures of development and concerns about gentrification around the start of V2V. The V2V strategies of facilitating investment and home-buying in Cluster C were similar to those used in Cluster B.

**Table 1:** Relative comparison of Community Development Clusters’ broader neighborhood conditions and characteristics in 2011 and 2019.^a^

	Cluster A		Cluster B		Cluster C	
	2011	2019	2011	2019	2011	2019
Population size	11,816*	9,376**	7,781*	6,093**	15,020*	17,099**
Median household income	$32,171	$32,833	$32,145	$37,328	$38,331	$53,664
% of population aged 16–64 year who are employed	45.6	53.6	40.1	50.4	57.3	76.2
% of households living below poverty threshold	21.4	18.2	28.8	34.5	5.5	6.5
Education among population aged 25–64 years						
% with less than a high school diploma	26.9	21.1	41.8	24.2	14.4	8.4
% with high school diploma and some college or Associate’s Degree	64.8	65.5	55.4	68.1	28.5	30.7
% with a Bachelor’s Degree or above	8.3	13.4	2.9	7.7	57.2	60.9
Racial/ethnic composition						
Non-Hispanic Black/African American	94.4	93.6	90.3	86.1	32.1	34.0
Non-Hispanic White	2.8	3.1	3.1	3.0	52.7	48.7
Hispanic	1.1	0.6	4.0	8.7	3.9	6.0
% Vacant properties	23.9	15.2	22.7	19.8	4.7	1.3
Median price of homes sold	$21,500	$81,340	$16,000	$120,000	$190,000	$240,000
Healthy food availability^b^ (2012, 2015)	9.75	7.98	10.1	9.7	13	11.2
Fast Food Outlet Density (per 1000 residents)	1.6	2.1	4.6	2.1	2.1	1.0
Liquor Outlet Density (per 1000 residents)	1.1	0.8	1.8	1.2	1.9	1.7
Crime rate (per 1000 residents)	54.6	54.3	73.5	84.6	99.5	87.7
Violent crime rate (per 1000 residents)	16.7	19.1	20.9	31.5	13.9	23.8

^a^Source: Baltimore Neighborhood Indicators Alliance (BNIA)-Jacob France Institute *Vital Signs* 2011 and 2019 report

^b^Defined by Average Healthy Food Availability Index

*Source: BNIA *Vital Signs* 2010 report

** Source: BNIA Vital Signs 2020 report

### Participant recruitment

We used maximum variation purposive sampling and recruited key informants across Clusters (n = 24). Beginning with key informants in DHCD who managed the implementation of V2V, we used snowball sampling to recruit additional participants involved in each of the three Clusters, including residents, local government officials, community-based organizations, and developers. We recruited 6 city officials, 6 developers, 3 representatives of community-based organizations, and 9 residents. Overall, long-term residents lived in their respective neighborhoods for a median of 20 years (interquartile range 14 to 35 years). City officials were positioned to speak on all three Clusters; 5 individuals were affiliated with Cluster A; 5 with Cluster B, and 8 with Cluster C.

### Data collection

We conducted semi-structured, in-depth interviews with key informants from December 2017 to June 2018. Interview guides were framed by the study’s research questions and elicited information about: key informant’s role in the Cluster, vacant housing and neighborhood conditions before revitalization, experience with V2V including perceived challenges and successes, and neighborhood changes including the community health impacts (see Supplementary materials). Guides varied slightly depending on the type of informant e.g., residents were asked about engagement with City agencies and City representatives were asked about engagement with other City agencies. Each interview included notetaking and audio-recording, with the informant’s permission, and ranged from one to two hours.

### Data management and analysis

Interviews were audio recorded and transcribed, and transcripts were uploaded to NVivo (QSR International, Doncaster, Victoria, Australia) for data management and coding. We drew from grounded theory approaches to analyze our data (Charmaz 2006) and conducted initial open coding on a small sample of transcripts, resulting in a comprehensive list of inductive codes. This first cycle of codes informed the development of focused codes, which were used to construct a codebook comprising inductive and deductive codes based on our broader research questions and the interview guide. Two coders used the final codebook to independently code all transcripts. There was high agreement in coding between the two coders, with discrepancies resolved through consensus. Using a constant comparative method, we examined similarities and differences, as well as relationships, between codes to identify emergent thematic patterns across interview transcripts. We further compared codes and themes by conceptually relevant attributes, such as respondent type (resident vs. government official vs. developer) and Cluster to identify patterns that differed by attribute. Data management, coding, and comparative analyses were conducted using Nvivo (QSR International, Doncaster, Victoria, Australia).

## Results

The results are presented in four sections. The first section examines how changes in the physical environment was perceived to be associated with health, reviewing the role of housing conditions and surrounding community amenities, including green spaces. The second examines changes in the social and cultural environment which can, in turn, impact levels of social cohesion and trust among residents and other stakeholders, ultimately influencing progress toward a shared vision of a healthy and revitalized community. The third section examines structural determinants vis-à-vis revitalization policies and practices that can generate or reinforce individual socioeconomic position, shaping people’s access to material resources and health. The final section examines structural determinants of community engagement and health and the role of neighborhood planning processes that can facilitate or hinder residents’ responses to neighborhood change.

### Changes in the physical environment and health

#### Housing conditions and health

Vacant housing revitalization was associated with improved housing conditions and a cleaner, safer neighborhood environment as homeowners moved into new housing, signaling more “eyes on the street” as noted by one resident:

“I used to have cookouts in the backyard and I stopped because it was infested with rats and the houses was boarded up. Now they done built three houses in the back. My son opened the door the other day…‘He said mom, it is so pretty out here now, so pretty.’” –Resident

Another resident similarly described noticeably less loitering and violence on the streets in Cluster B, while in Cluster A, respondents noted that safety and crime were still major concerns, despite some reported improvements with regard to site control and illegal dumping. Left unaddressed, chronic exposure to crime and violence were described as contributors to endemic levels of mental health challenges, including stress, trauma, low self-esteem, and hopelessness.

Further, mental health conditions were detrimental to residents’ ability to maintain a livelihood, creating a feedback loop that could exacerbate and reinforce mental illness and risk of vacant housing. Several respondents further emphasized that health is achievable when, in addition to safe and affordable housing, the basic need for gainful employment and economic security is met. Residents described how a lack of educational and economic resources may contribute to a sense of hopelessness and drive trauma, illicit drug use, stress, and depression. While outside the traditional purview of neighborhood revitalization, Cluster B partnered with a local non-profit organization to create jobs and hire residents to deconstruct vacant homes. This was described as having the immediate effect of building goodwill for the revitalization project and over the long term, hired residents also gained marketable job skills.

#### Community amenities and health

Residents and city officials also indicated an absence of safe outdoor spaces for people to socialize and children to play prior to V2V. Therefore, the transformation of vacant lots into green space, following demolition, was described as enhancing walkability and providing opportunities to support residents’ physical and mental wellbeing:

“The density [of vacant houses and buildings] in that footprint was pretty stark…we did some spot demolition in conjunction with the wishes of the community, so created a lot of green space…which has to be healthier for people.” –City Official

Beyond the implementation of greening strategies, respondents in all three Clusters described the need for community amenities, which were perceived to be equally imperative for reinforcing health but constrained by the limited scope of V2V. Some respondents associated a high density of liquor stores with criminal activity and violence, producing additional mental health and safety risks to residents. Additionally, a lack of access to healthy, affordable food options and health care was also reported to negatively affect communities’ wellbeing. Food options largely comprised corner stores with few choices for fresh produce, and the nearest supermarkets were not easily accessible. Residents expressed strong interest in diversifying food choices in the community—a desire also acknowledged by city officials and developers, yet one that remained unaddressed.

“…That’s a big problem in the city. This neighborhood is notorious for not having great access to supermarkets…[But] it wasn’t one of these things we could do that much about. –Developer

### Changes in the social and cultural environment and health

#### Social fabric and health

Vacant housing revitalization was perceived by residents to spur changes in the social and cultural identity of respective communities with demographic shifts impacting the overall fabric of a neighborhood. Prior to revitalization, residents in the three Clusters were predominantly older adults or working-class families and renters. Reported increases in racial and economic diversity were particularly evident in Clusters B and C. Residents of these clusters described changes in racial, generational, and household size, noting that new residents moving in were predominantly white, of the Millennial generation, and childless. One resident described how the streets of her neighborhood used to be filled with the voices of children playing whereas today that neighborhood atmosphere no longer exists due to the lack of families on the street. Developers also described similar changing demographics, with one developer noting this shift is not what was intended when they were working to revitalize properties in Cluster C. This developer described how they intended for the new market to target existing renters and prospective homebuyers who had middle-class incomes. Instead, this developer reflected on how most buyers were young Millennials:

“The goal, we originally thought that our market would be people making between 45 and 65 thousand, able to afford a $200,000 house and that the buyers would be renters, single, female heads of households, who are renting for as much money as the mortgage would cost. We thought we’d have maybe 80% that market and then 20% young Millennial workers…It’s the reverse.”—Developer

In contrast, some residents believed that revitalization efforts were never intended for those who were already living there, including both homeowners and renters. One resident from Cluster C described how residents were approached to buy homes elsewhere outside of the cluster but were not provided the opportunity to obtain homeownership of a redeveloped home within the cluster. Another resident described how they saw no impacts from revitalization other than moving existing residents out of the neighborhood, which should not be considered a positive outcome of neighborhood revitalization.

“I think that some people that was here are not going to be able to...afford to get back in here…They got pushed out when the development came…It’s a healthy community, a safer community, but it’s not going to be an affordable community for the people who used to be able to afford to live here.” –Resident

#### Social cohesion between new arrivals and long-term residents

Long-term residents also placed high value on building community and supporting families, particularly advocating for more child-centered neighborhood revitalization plans, such as investing in building playgrounds and co-creating initiatives with youth. However, some of the newer single, adult residents had other preferences regarding the design and use of neighborhood spaces to fit their needs and lifestyles. Participants noted such differences sometimes led to disagreements between long-standing and newer residents at community association meetings, provoking reflections about whose needs were being prioritized and how to remediate conflicts moving forward.

“It’s those conversations about race. It’s the conversations about class…We have so many types of people who live here…Even though someone may talk about being in their $400k home…You got to be able to engage with the people across the street from you who live in a subsidized housing. You can’t just think, because you purchased this, everybody’s going to do what you say and what you want. It just doesn’t work like that.” –Resident

While most supported V2V’s implementation of greening strategies, conflicts arose concerning what the green space was for and who the primary users would be. From the perspective of a developer, green space benefited community health by creating opportunities for physical activity. Meanwhile, respondents recounted debates in Cluster B and C among residents about creating a dog park to accommodate dog owners in the neighborhood, many of whom had recently moved in, as opposed to accommodating children with play space, a preference of long-term residents. These differences in values challenged community norms of prioritizing child-centered amenities and how people (versus pets) engage in space. One resident explained the situation:

“We were like, ‘We want to build a park.’ So, now our community is debating over a dog park. Where [is] the people park? We’ve been asking for a park for kids for forever. Now you want a dog park on it.“ –Resident

Cohesion and trust among residents were further threatened when developers and newer residents failed to respect and acknowledge the history of community work that had gone into restoring the neighborhood. Long-time residents called for more inclusive communication and authentic appreciation of existing social norms and community identity in order to better align revitalization plans with *all* residents’ values.

“You have people that are starting to separate and have no respect for the people who did do things here [before] they came here. Mind you, if you bought a house here and you do live here… you’ve got to have some type of respect for the people that put their blood, sweat, and tears in this community.” –Resident

### Structural determinants of material resources and health

#### Transfer of vacant home ownership and revitalization opportunities

The regeneration of vacant housing required a formal transfer of the property to an investor or developer. DHCD heavily relied on two mechanisms to expedite this transfer: eminent domain, which enabled direct transfer of the property to the City, and receivership, which enabled DHCD to choose a “receiver” to sell, renovate, or destroy the vacant property. DHCD then issued requests for proposals from prospective developers, or the property was directed to auction (in the case of receivership). Ultimately, buyer qualifications made it challenging for smaller developers (including non-profit developers, individual buyers) to acquire homes and participate in V2V (Jacobson 2015). The financial capital required of a receiver (at least $90,000) in conjunction with the City’s preference to redevelop larger footprints led to inviable plans proposed by developers who wished to invest in smaller projects. For example, one non-profit community development organization described how they had originally proposed a very small plan to regenerate a footprint of just three vacant homes, to which DHCD responded a larger demolition and rebuild would be preferred.

#### Benefits of homeownership incentives

Developers described homeownership as a means to increase residents’ economic resources and improve their overall quality of life. Yet, some residents noted unequal distribution of these benefits. Once a vacant home was restored, incentives for homeownership were reportedly unequally accessible to all prospective homebuyers, particularly existing renters in the neighborhood. For example, incentives offered by V2V were coupled with additional offers through a homebuyer’s employment with neighboring private and public institutions that ultimately contributed toward the down payment of a new home – anywhere from $17,000 to a high $36,000 – helping to reduce monthly payments. These pathways to homeownership were perceived by residents to encourage new and wealthier first-time homebuyers, excluding long-term residents and renters in the neighborhood who did not share access to the same financial incentives. Developers also reportedly benefited as these incentives enabled them to market redeveloped homes at more profitable prices.

While employer benefits paired with V2V successfully created some mixed-income housing, long-term residents expressed shock that homes sold for $200,000 to $400,000 in their neighborhoods following revitalization investments from the city and developers, creating challenges for lower-income residents. One resident described how rising housing prices resulted in rising rents within their neighborhood, but little support was offered to residents from the city to deflect challenges with housing affordability. Others who were working class and long-term renters also described concerns of displacement and wealth exclusion, meaning the wealth acquired through homeownership benefited individuals from outside the neighborhood rather than those from within the community. Some city officials and developers discussed a rent-to-own model, land trusts, and community benefit agreements to create opportunities for renters to build wealth through homeownership. However, no such mechanisms were reported to have come to fruition. Instead, city officials and developers commonly mentioned these models in reflection as ways to promote more diversity in wealth generation. Residents described a need for mechanisms and incentives that invest in low-income residents and long-standing renters by providing pathways to homeownership through V2V.

“We had all of these vacants, and we have all of these renters. They were still living here. Not one renter has evolved to a homeowner. You can become a homeowner, but you won’t be one here. They’ll give you a voucher…to be a homeowner…somewhere else.” –Resident

### Structural determinants of community engagement and health

#### Revitalization plans’ (mis)alignment with community priorities

Neighborhood planning was most described as a mechanism for residents to voice their values and priorities and to engage with developers and city officials implementing V2V. However, some developers expressed mixed opinions on the true utility of these plans, which were perceived as often lacking clear directives for developers who did not understand neighborhood planning processes. One developer noted that under-developed neighborhood plans contributed to misalignment between the city’s revitalization plans, developer interpretations, and residents’ priorities.

“…[neighborhood plans are] a typical product of planners who don’t understand development, and there are too many developers who don’t understand planning. Trying to bridge those gaps is very, very difficult…It just meant a lot of night meetings and frustration…It just isn’t very useful.” –Developer

Misalignment between revitalization plans and resident’s priorities was further demonstrated in the planning of green spaces surrounding revitalized homes. Local government officials and developers described good intentions for the development of green space, yet in one Cluster, failed to meet the terms of the community benefit agreement. In this case, the respective community master plan had included recommendations for community benefit agreements to be incorporated in all development projects, including those that involved regenerating vacant housing. As such, the developer worked with the builder and community residents to develop a community benefit agreement around the development of green space. However, the green space was reportedly not maintained nor designed it in alignment with residents’ wishes. Notably, many long-term residents spoke to the changing identity in their neighborhoods where V2V implementation had progressed further, with some who believed the outcomes of revitalization were never intended for them. For example, one long-time resident in Cluster C exhibited a growing sense of distrust and exclusion:

“The second biggest obstacle now in the neighborhood is parks…There’s no neighborhood park with swings…what normal kids would have. They have properties that are designated park land, but…there’s no infrastructure on them...It’s called […] Park, and it has nothing in it.” –Resident

#### Unequal burden and engagement practices between and within stakeholder groups

City officials were described as reactive to communities’ concerns and focused on marketing the V2V initiative, while community organizers were perceived as proactive, often carrying the responsibility of building and maintaining responsive relationships with local government officials and developers. In the same vein, city officials and developers affirmed their reliance on community leaders to mobilize other residents around revitalization activities, and one resident noted that local government officials were more likely to respond to pressures resulting from repeated criticisms by various residents over an issue or concerns raised collectively by a community association. Across all stakeholders, communicating about V2V with the support of a community leader was believed to be instrumental in advancing the city’s revitalization agenda while facilitating relationship-building with residents. Although community leadership was necessary to foster inclusive decision-making, it was not sufficient in the context of historic economic disinvestment. Diminished cohesion among different community leaders and residents in Cluster A, for example, resulted in neighborhood planning disagreements (e.g., how to use available funding) making it challenging to work collectively and delayed progress in revitalization efforts. Respondents from Cluster A described a patchwork of different community associations, organizations, and leaders who often had conflicting agendas and struggled to develop harmonized goals and strategies. Additionally, DHCD was described as regularly breaking revitalization promises within Cluster A, further entrenching disparities in access to resources. The lack of cohesion and trust among community leaders and members in this setting likely created unique barriers to achieving collective efficacy.

“Nobody wanted to work together ‘cause they were scared somebody was gonna get more money than them...we always had that crab mentality…versus [other neighborhoods] where they would work together...they’re very selective [about] who they choosing to play in the sandbox…everybody plays a part of not working collectively.” –Resident

## Discussion

This study adds to the literature on vacant housing and the need for more than remedies to the physical environment to maximize health. Our findings demonstrate how vacant housing revitalization influences the physical environment, social environment, and structural determinants of material resources and community engagement that can ultimately influence residents’ physical, mental, and social health. Because housing disparities are rooted in structural inequalities, how policies, practices, and processes in vacant housing revitalization moderate pathways for residents to respond, adapt, and thrive alongside neighborhood changes is consequential for health and health equity.

First, this research builds on existing literature linking neighborhood change and health. Consistent with prior studies, we found improvements in perceived safety and mental health resulting from the revitalization of vacant homes and vacant land into green space (following the demolition of vacant housing). Restoring vacant housing and greening strategies have been previously documented to promote physical and social activity and decrease depression (South et al. 2018). However, we found these health benefits manifested in different ways in the context of V2V especially among residents in Clusters that had experienced a greater degree of neighborhood change (i.e., Clusters B and C) since the start of the revitalization initiative nearly a decade ago. For example, while stakeholders overall supported the development of green space for physical health, lack of attention to the social and cultural significance of green space, such as its purpose and who the primary users were intended to be, negatively impacted residents’ mental health. This is supported by emerging scholarship on associations between green gentrification and long-time residents experiencing social exclusion and a lower sense of community (Jelks et al. 2021). Meanwhile, in the Cluster that had experienced a lesser degree of neighborhood change (i.e., Cluster A), long-time residents experienced a slightly different, yet still impactful, sense of social exclusion as local government officials and developers failed to meet the terms of the community benefit agreement by not properly maintaining the green space nor designing it as residents had expected.

Second, outcomes of this study begin to highlight the need for vacant housing revitalization to consider factors beyond the built environment to prevent from counteracting the benefits of an initiative like V2V. We provide a more nuanced understanding of the social dynamics influencing how vacant housing revitalization can engender social and cultural environmental change that is often linked with gentrification, and ultimately health. Access to supportive social networks in promoting health has been well-documented (O’Malley et al. 2012; Fiori et al. 2006; Kawachi and Berkman 2001). Residents and city officials similarly described residents’ social networks serving as an accountability system and a means of organizing around shared goals related to building a revitalized and healthy community. Further, more recent scholarship on gentrification argues for a need to better elucidate the “affective, emotional and material *rupture*” that occurs between people and place as neighborhood environments undergo change (Elliott-Cooper et al. 2020). Upon a closer examination of social networks in the context of vacant housing revitalization, our results indicate that the degree of support produced and facilitated by social networks depended on the degree of cohesion and trust among both existing in-group relationships (i.e., between residents within a community) as well as between-group relationships (i.e., between residents, developers, and city officials). Importantly, we found that as revitalization activities unfolded and changed neighborhood demographics, existing relationships were also challenged, especially when community social norms were called into question. For example, following demolition of a vacant home, the development of green space—though well intended—highlighted tensions that arose between long-term residents and new arrivals or between homeowners and renters, due to conflicting social norms, values, and preferences. As described earlier, this experience is not unique to this context, and more recent literature has also referred to this phenomenon as “eco-gentrification” (Richards, 2020).

We further learned that residents’ capacity to respond and adapt to neighborhood changes benefited from the presence of community leadership. In other words, strong leadership resulted in a greater likelihood of mitigating conflicts, prompting city officials and developers to address residents’ concerns, and building consensus around a shared vision and value system for a healthy and revitalized community. For example, in Cluster C, neighborhood leaders actively planned community events for all residents, new arrivals and long-term, to interact and engage with one another. This relationship-building helped foster cohesion and mobilize residents to advocate for strategies to address neighborhood concerns. Cluster A, on the other hand, suffered from a dearth of strong leadership and internal conflicts between community groups, stifling opportunities to develop the unity and stability necessary to advance a shared agenda. It is important to note that aside from community leadership, developers and city officials can also play an important leadership role in shifting the norms around engagement practices. Therefore, efforts to revitalize neighborhoods require not only the engagement of local government and developers, but also a commitment to foster existing and new relationships with and among residents in the community. Without proactive and inclusive relationship-building, low-income households and renters are more likely to be excluded from some of the economic benefits, while facing pressures related to physical and cultural displacement (Cornelius and Wallace 2010; Pastor and Morello-Frosch 2014; Raja et al. 2021; Sanchez and Brenman 2012).

Importantly, the policies, practices, and processes of V2V described in this study ought to be situated within the broader context of contemporary city planning that is dominated by neoliberal urban development and political regimes. From top-down, market-driven planning and policy decisions, such as V2V’s focus on neighborhoods with “emerging markets” rather than areas with the greatest need, to V2V’s reliance on for-profit developers or quasi-public economic development agencies for funding and resources (Hackworth 2011; Baeten 2017), our findings begin to illustrate some of the ways in which core components of V2V reflect neoliberal origins. Growing literature reports on the detriments of neoliberal revitalization programs, including outcomes such as gentrification, reduced affordable housing, and the erosion of community cohesion and collective interests in favor of privatism (Hackworth 2011; Garboden and Jang-Trettien 2020; Pill 2020) that can ultimately harm community health and wellbeing, including affects on mental, social, and physical health (Smith et al. 2020; Taylor 2018). In spite of these structural challenges, however, we also observed examples of community leadership and social cohesion that challenged the norms of revitalization decisionmaking processes and pushed the interests of long-term residents to become an integral part of neighborhood planning activities (Stone 2005, 2006).

Ultimately, vacant housing revitalization will produce the greatest health benefits if plans and processes are co-produced and implemented with community members, including long-term and lower-income residents (Mattessich and Rausch 2014; Watson 2014). In addition, because Black and other residents of color are more likely to experience a higher concentration of vacant housing in their neighborhoods, careful consideration of policies, practices, and processes that apply an asset-based approach is important for elevating local resident leadership and ownership to drive revitalization initiatives, which in turn would more directly benefit residents and their wellbeing (Kretzmann and McKnight 1996). Developers and housing officials can begin to successfully improve health and foster equitable community benefits by learning about the social fabric among residents and leaders before implementating revitalization plans. A narrow focus on the neighborhood physical environment i.e., restoring vacant housing, runs the risk of excluding local assets, such as homebuyers and nonprofit developers, while promoting real estate speculation by private developers who often are less embedded in the community. In this study, we reported on the complex and dynamic nature of social networks that influenced progress toward a shared vision of a healthy and revitalized community. At a minimum, local governments should understand different social structures and levels of trust that can vary across neighborhoods and be prepared to adapt revitalization processes to the needs of each community accordingly. For example, the presence of a community “backbone organization,” such as an established neighborhood group or community-led organization, can ensure residents’ needs are adequately represented and support shared decision-making in revitalization plans between residents, local government decision-makers, and developers (Flood et al. 2015; Kim et al. 2020). In neighborhoods where social fractures may exist, local governments should invest time and space for relationship-building and joint decision-making. One example strategy is for local governments to establish shared governance by creating an advisory group comprising residents, developers, and local government representatives. Once an advisory group is identified, it is valuable to have members clearly articulate common values, goals, objectives, and outcomes related to revitalization through a visioning process. Visioning can be facilitated by aligning neighborhood plans with revitalization plans and using public engagement methods, such as charettes, listening sessions, and workshops, that invite the broader resident community into these conversations and shared decision-making process (Lowe 2008). More integrated and inclusive processes can minimize “zero-sum” approaches and foster planning that is responsive to varying needs of residents, such as multifunctional green space for individuals across life-course stages and with diverse preferences in the use of green space (Douglas et al. 2017).

Lastly, we build on prior literature on the structural determinants of homeownership and health by documenting revitalization pathways involved in the unequal distribution of material resources and benefits (Long and Caudill 1992; Turner and Luea 2009; Shapiro 2006). Respondents across Clusters acknowledged economic conditions that are consequential for health, most notably long-term residents’ chronic exposure to exclusionary wealth-building opportunities that reportedly contributed to poor mental health. To avoid counteracting the goals of vacant housing revitalization initiatives, endeavors such as V2V can aim to improve the quality of life of long-term residents more expansively. Investing in long-term residents’ homes with opportunities for parallel structural improvements or pathways that enable renters to evolve into homeowners is one starting place. Additionally, residents described wealth generation in varied forms, including education and employment opportunities. While building schools and creating jobs may not fall within the traditional purview of vacant housing revitalization, urban regeneration efforts aspire to promote community health. Therefore, within its own realm of influence, vacant housing revitalization has a role in engaging with the community system more holistically by creating opportunities to hire talents and labor from within the community, such as in Cluster B, and aligning revitalization activities with other essential health needs. For instance, prior research has documented how perceptions of the local school system can influence residential stability by attracting and retaining families or promoting residential turnover (DeLuca and Rosenblatt 2017). In a similar vein, healthy food retail is a valuable asset to a community, not only by providing neighborhood residents with access to nutritious food but also fostering living wage jobs and economic development. Including investments in healthy food retail as part of a more comprehensive vacant housing revitalization strategy could serve as an anchor to attract additional businesses and further bolster community benefits (Silver et al. 2017).

Considering the present findings, this research is not without limitations. The effects of V2V on health outcomes are challenging to isolate, and other sociopolitical factors beyond the V2V initiative during this study period may have contributed to perspectives on the observed changes. However, results from this study offer a proof-of-concept to begin unpacking the dynamic and complex relationship between physical, social, and economic dimensions affected by neighborhood revitalization initiatives. Although we reached theoretical saturation, it is possible that a larger sample including more representation from residents, such as additional perspectives of newer residents who moved into the Clusters, may have resulted in different viewpoints. In future research, a larger representative study focused on residents, including experiences of residents that identify differentially by race and ethnicity, might further elucidate the relationships between V2V and health outcomes. Recall bias and social desirability are additional concerns that we aimed to minimize by providing respondents with assurances about confidentiality and no wrong opinions; acknowledging diverse experiences and challenges in all communities; and probing for more information, stories, or examples. This study centered on the regeneration of vacant housing, so generalizability of our findings to other neighborhood revitalization initiatives may be limited. Future research is needed in other cities and contexts to gain a richer understanding of the beneficial and harmful impacts of vacant housing revitalization on health.

## Conclusion

Vacant housing revitalization can provide community benefits in many forms, including improved housing quality, mixed-income housing, and green space. However, residents’ response and adaptation to neighborhood changes, especially among long-term residents, are essential for maximizing health benefits and reducing health inequalities. Like the physical environment that requires design, construction, maintenance, and monitoring, the social environment, too, requires the same – if not more – attention and planning to redress inequities in housing and health. Our findings uncover nuances in some of the policies, practices, and processes of vacant housing revitalization that can moderate social environmental changes impacting health, both positively and negatively. The health benefits associated with improved housing structures, perceived safety, and access to green space were diluted by detriments to residents’ social and cultural environments, particularly experienced by long-time residents. Ultimately, vacant housing revitalization will produce more favorable physical and mental health outcomes with the establishment of shared governance structures and resources (Mattessich and Rausch 2014; Watson 2014; Jutte et al. 2011).

While V2V was initially created and implemented under the leadership of Mayor Rawlings-Blake (2010–2016), the program presently remains a part of the City’s strategy to address housing vacancy and blighted properties in Baltimore. Currently, Mayor Scott has made it a priority to build on past rehabilitation programs and develop a holistic approach to eliminating vacant housing, which includes a $100 million investment from the American Rescue Plan toward Community Development Clusters (French 2022). At a minimum, local governments officials should be clear on different social networks and levels of cohesion that can vary across neighborhoods and be prepared to adapt engagement practices to the needs of each community. Residents must also become familiar with the role and functions of local governments and developers and be willing and prepared to engage. Going forward in urban regeneration, pathways to *retain* and strengthen the social environment while revitalizing the physical environment can be promising to achieve a healthy community in its fullest sense.

## Electronic supplementary material

Below is the link to the electronic supplementary material.

Supplementary Material 1: Interview guide.

## Data Availability

The data generated during the current study are not publicly available because study participants who participated did not consent to the data being made publicly available.
