# Supplementary material for: Revisiting revitalization: exploring how structural determinants moderate pathways between neighborhood change and health
Source: Int J Equity Health. 2022 Nov 18;21:165. doi: 10.1186/s12939-022-01771-9 (PMC9675168; doi:10.1186/s12939-022-01771-9)
Supplement: Supplementary file 1 — Supplementary Material 1: Interview guide. [file 12939_2022_1771_MOESM1_ESM.docx]

**Supplementary Material**

*Interview guide*

Interview guide questions were chosen and ordered as appropriate to each type of informant and the flow of the conversation:

**Background**

## Please tell us a bit about yourself: confirm your name, position, title, and how long you have held your current position

## Can you explain what you do (e.g., outline current/previous roles and responsibilities as they relate to housing in Baltimore?)

## Can you describe your organization (e.g., from your perspective what is its mission, what are its goals, what is its recent history?)

## Can you tell us a bit about this neighborhood?

**Vacants to Value (V2V) Initiative**

1. Please describe conditions in the neighborhood before the V2V initiative.
2. Please tell me the story of the V2V Initiative in your neighborhood.
   1. PROBE: When did the Initiative start in your neighborhood?
   2. PROBE: What interactions (direct or indirect) have you had with the Initiative?
   3. PROBE: To what extent have you interacted with City agencies (e.g., with code enforcement, 311, others)?
   4. PROBE: What have been some challenges? Strengths?

**Community Health**

1. Can you please describe what your community’s health means to you? When you hear ‘community health,’ what do you think of?
2. In what ways do you think vacant properties have impacted your health or the health of others (e.g., physical health, mental health, safety, etc.) in your community?

**Neighborhood Change**

1. Can you please describe changes, if at all, that you have experienced in your neighborhood since the V2V Initiative (e.g., on community members, local economy, housing, businesses, safety, etc.)?
2. Since V2V, how has the number of vacant, blighted, or abandoned properties changed? Improved, worsened, or remained the same?
3. Since V2V, what changes, if at all, have you noticed in your health or the health of others in your community (e.g., physical health, mental health, safety, etc.)?
4. Since V2V, can you please describe how safety in your neighborhood has changed? Has it increased, decreased, or remained constant in its frequency or intensity?
5. Since V2V, how has recreation/green space available to children and adults changed, if at all? Have there been any new parks added to your neighborhood?
6. Since V2V, what changes, if at all, have there been in neighborhood businesses (e.g., food retailers)?
7. What else have we not covered that you think is important for us to know related to how the V2V initiative has affected your health or the health of others in your community?
